# Abbreviation of preoperative fasting and malnutrition: impact on cost-effectiveness of surgical patients

**DOI:** 10.1590/0100-6991e-20253776-en

**Published:** 2025-06-19

**Authors:** WESLEY SANTANA CORREA ARRUDA, DIANA BORGES DOCK-NASCIMENTO, JOSÉ EDUARDO DE AGUILAR-NASCIMENTO

**Affiliations:** 1 -Universidade Federal de Mato Grosso, Pós-Graduação em Ciências da Saúde da Faculdade de Medicina - Cuiabá - MT - Brasil; 2 - Universidade Federal de Mato Grosso, Faculdade de Nutrição - Cuiabá - MT - Brasil; 3 - Universidade Federal de Mato Grosso, Departamento de Clinica Cirúrgica do Hospital Universitário Júlio Muller - Cuiabá - MT - Brasil

**Keywords:** Perioperative Care, Cost-Effectiveness Analysis, Health Care Costs, Enhanced Recovery After Surgery, Fasting, Malnutrition, Assistência Perioperatória, Análise de Custo-Efetividade, Custos Hospitalares, Recuperação Pós-Cirúrgica Melhorada, Jejum, Desnutrição

## Abstract

**Introduction:**

Abbreviating preoperative fasting improves clinical outcomes, such as reducing hospital stay, morbidity and postoperative mortality. However, there is a lack of data regarding the reduction of hospital costs. Therefore, the objective of the research was to analyze whether reducing preoperative fasting time with the provision of carbohydrate-rich liquid has an influence on hospital costs for surgical patients.

**Methods:**

Retrospective data were collected from patients undergoing surgical procedures at a University Hospital in 2019. The main outcome variable investigated was cost hospital in reais. Data such as gender, age, type of surgery, length of stay, nutritional data, and outcomes were also collected. Next, a comparative analysis of the variables was carried out in relation to patients who followed the preoperative fasting abbreviation protocol, with liquid rich in carbohydrates, and those whose protocol was not applied.

**Results:**

The mean (± standard deviation) fasting time of the study participants was 267.92±89.8 (range: 120-605) minutes in the group that shortened the fast and 768.6±247.8 (150 -1244) minutes in the group that did not perform the abbreviation (p<0.01). In relation to hospital costs, it was observed that patients who shortened their fasting had a lower average cost than those who did not shorten it (R$ 3,245.37±4,157.5 vs R$ 10,897.39±16,701.3; p< 0.01). They were shown to be significantly associated with higher cost, malnutrition and prolonged preoperative fasting.

**Conclusions:**

According to data from this study, shortening preoperative fasting reduces hospital costs. Corroborating prolonged fasting, malnutrition also makes hospitalization more expensive.

## RESUMO

**Introdução:**

A abreviação do jejum pré-operatório melhora desfechos clínicos, tais como redução de tempo de internação, morbidade e mortalidade pós-operatória. Porém há escassez de dados em relação à redução de custos hospitalares. Portanto, o objetivo da pesquisa foi analisar se a redução do tempo de jejum pré-operatório com a oferta de líquido rico em carboidratos tem influência nos custos hospitalares de pacientes cirúrgicos.

**Métodos:**

Foram coletados dados retrospectivos de pacientes submetidos a procedimentos cirúrgicos em um Hospital Universitário no ano de 2019. A variável principal de resultado investigada foi o custo hospitalar em reais. Também foram coletados dados como sexo, idade, tipo de cirurgia, tempo de internação, dados nutricionais, e desfechos. Em seguida foi feita a análise comparativa das variáveis em relação aos pacientes que seguiram o protocolo de abreviação do jejum pré-operatório, com líquido rico em carboidratos, e aqueles onde o protocolo não foi seguido.

**Resultados:**

A média (± desvio padrão) de tempo em jejum dos participantes do estudo foi de 267,92±89,8 (intervalo: 120-605) minutos no grupo que abreviou o jejum e 768,6±247,8 (150-1244) minutos no grupo que não fez a abreviação (p<0,01). Em relação aos custos hospitalares, observou-se que os pacientes que fizeram a abreviação de jejum apresentaram custo médio menor que aqueles que não abreviaram (R$ 3.245,37±4.157,5 vs R$ 10.897,39±16.701,3; p<0,01). Mostraram ser significantemente associados ao maior custo, a condição de desnutrição e o jejum pré-operatório prolongado.

**Conclusão:**

A abreviação de jejum pré-operatório diminui custos hospitalares. Corroborando ao jejum prolongado, a desnutrição também encarece a internação.

## INTRODUCTION

Based on evidence, new protocols for perioperative care of surgical patients, such as the Enhanced Recovery After Surgery (ERAS)^1^, began to emerge in the literature, seeking the use of scientifically proven routines to the detriment of traditional ones, generally considered outdated^2^.

In Brazil, the ACERTO Project (ACEleração da Recuperação Total Pós-operatória)^3^, created at the Júlio Muller University Hospital (HUJM), of the Federal University of Mato Grosso (UFMT) in 2005, is a multimodal protocol based on ERAS, which, among other measures, institutes the shortening of the preoperative fasting period^4^ ^,^ ^5^. 

Traditionally, patients are fasted for 12 to 24 hours before elective surgical procedures, with the aim of ensuring gastric emptying and reducing the risk of emesis and aspiration at the time of anesthesia induction^6^ ^,^ ^7^. However, studies question this practice, as this time is usually long^8^ ^,^ ^9^, leads to stress, discomfort, malnutrition, and contributes to worse clinical outcomes^10^.

When the individual is subjected to the fasting process, even with the reduction of basal energy expenditure, the energy requirement in the tissues for vital functions persists, and thus, several metabolic reactions, such as glycogenolysis, proteolysis, and lipolysis, occur to maintain blood glucose and energy supply^3^ ^,^ ^11^ ^,^ ^12^. 

The period of exacerbated fasting, added to the trauma imposed by the operation, implies an increase in catabolic hormones such as cortisol and glucagon, inflammatory response, and secretion of catecholamines^11^, with catabolism as the main consequence, further increasing the breakdown of energy reserves^12^. 

Thus, the abbreviation of fasting with the use of a carbohydrate-enriched beverage (12.5% maltodextrin solution) up to two hours before the operation can reduce the organic response to surgical trauma, reducing the insulin response^13^ ^,^ ^14^, the inflammatory reaction^15^, impacting on better clinical outcomes such as a reduction in hospitalization time and postoperative morbidity and mortality^16^ ^-^ ^19^, and has been recommended by guidelines for surgery^20^, nutrition^18^, and anesthesiology^21^.

However, there is no published data on the effectiveness of shortening preoperative fasting in reducing hospital costs. The analysis of the population’s spending on medical care is common in other countries, but rare in Brazil. This analysis can be a powerful tool for the elaboration of public policies.

Therefore, the present study aimed to analyze whether the reduction of preoperative fasting time with the supply of carbohydrate-rich liquid has an influence on hospital costs of elective surgical patients in a University Hospital.

## METHODS

This cross-sectional study was submitted to, and approved by, the Ethics in Research Committee (CEP) of the Federal University of Mato Grosso in 2020 (CAAE number 27330619.9.0000.5541/2020). We collected retrospective data from the electronic medical records of elective patients undergoing surgical procedures at the General Surgery Service (Department of Surgery) of the Júlio Muller University Hospital of the Federal University of Mato Grosso - MT, Brazil, between January and December 2019, the last year of normal operation of the service before the Covid-19 pandemic. 

We excluded patients whose medical records without data on the date of hospitalization, surgery, or outcome, and when it was not possible to find the time of surgery due to lack of surgical report. We also excluded patients whose information on the abbreviation of fasting could not be obtained.

The main outcome variable investigated was the cost in Brazilian reais. The secondary variables were gender, age, nutritional data from the Subjective Global Assessment (SGA), outcome (discharge or death), use of nutritional therapy, presence of systemic arterial hypertension (SAH), diabetes mellitus (DM), infections and complications, smoking, alcoholism, and shortening of fasting.

SGA was collected from the Clinical Nutrition medical records, through an assessment by a nutritionist in the sector, based on hospitalization data. The SGA then classified nutritional status as well nourished (SGA A), nutritional risk/moderate malnutrition (SGA B), and severe malnutrition (SGA C)^22^.

We considered nutritional therapy the use of oral supplementation, enteral nutritional therapy, or parenteral nutritional therapy at some point during hospitalization. After reading the medical records, we also recorded whether the patient had infections at any time during hospitalization or complications, such as abscesses, fistulas, dehiscences, and eviscerations. We collected data on diagnosis and type of surgery. Subsequently, we included the types of surgery in seven groups: digestive tract, urological, abdominal wall, head and neck, plastic, vascular, and others.

For the abbreviation of fasting, we searched for the item “surgical preparation” in the medical prescription and the time it was prescribed. The surgical preparation in the hospital is as recommended in the ACERTO protocol, composed of clear liquid without residues with 12.5% maltodextrin. The total amount of liquid offered is 400 mL six hours before the scheduled start time of surgery, and 200 ml two hours before. 

For patients without prescribed fasting abbreviation, we considered the time of the last meal as 10 p.m. on the day before surgery, the approximate time when supper is served. 

We subdivided the group of patients with abbreviated fasting into those in whom this time was actually reduced and those who, even with the use of surgical preparation, fasted for a long time due to a delay in surgery. For this, we adopted five hours as the cut-off point, based on the initial results of the implementation of the ACERTO protocol in 20064, as the average fasting time after the interventions.

For the daily hospitalization, we adopted the count of days in the period from the date of surgery to the date of outcome, which was classified as hospital discharge, transfer (from clinic or hospital), or death.

Thus, the cost of one day was multiplied by the number of days of hospitalization in the postoperative period.

### Cost analysis

Data on hospital costs were provided by the Evaluation and Controllership Sector of the Júlio Muller University Hospital. The cost accumulation method was used according to NBCT 16.11 - Public Sector Cost Information System^23^ ^,^ ^24^. This method allows an indirect calculation of the daily cost of patients as follows. To obtain the average cost of hospitalization per patient per day, the total costs of hospitalization in the ward of Surgical Clinics were divided by the average annual patient/day. Regarding the calculation of the average cost per number of hospitalizations, we divided the total costs of hospitalizations in the Surgical Clinics by the number of hospitalizations performed in each period. Finally, the value of the average cost of hospitalization per night consisted of dividing the total costs of hospitalization in the Surgical Clinic by the number of nights in the period.

For the purpose of calculating the average cost of hospitalization in the surgical clinics of the HUJM, we used the following data: (1) report on the output of products by sector issued by the MV 2000 inventory control system; (2) laboratory and imaging report issued by the MV 2000 exam billing system; (3) report on the movement of hospital admission authorizations (AIH) - reduced forms and rejected AIH issued by the DataSUS/Tabwin system; (4) data sheet of civil servants provided by the human resources unit of HUJM; (5) personnel data of employees in the single legal labor regime (RJU) provided by the HUJM settlement and payment of expenses unit; (6) working hours available on the HUJM website; (7) information on the amount of equipment in the operating room provided by the person in charge of that unit; (8) data from the clinical engineering contract, as well as footage of the hospital areas of the HUJM made available by the logistics and infrastructure division; and (9) information on accommodation costs obtained by the monitoring panel of hospital accommodation indicators and made available by the hospital accommodation unit. Thus, we obtained the amount of R$ 1,442.86 for the daily cost of a patient operated on in our ward.

### Data analysis

We evaluated continuous variables for normal distribution using the Kolmogorov-Smirnov (K-S) test, and for homogeneity of variances using the Levene test.

For univariate analysis in continuous data with Gaussian distribution, we used the Student’s t-test, and when the data distribution was not homogeneous, we used the non-parametric Mann-Whitney and the Kruskal-Wallis tests. For qualitative variables, we applied the chi-square and Fisher’s exact tests. A statistical significance limit of 5% (p<0.05) was established. For multivariate analysis, we employed a linear regression model, including variables that in the univariate analysis presented p<0.20. 

The results were expressed as mean, followed by standard deviation (SD), or even median and range, when appropriate. All calculations were performed using the SPSS statistical package version 20.0

## RESULTS

Of the 1,064 patients admitted to the surgical clinic in 2019, 232 were excluded from the series due to lack of data in the medical records. Thus, 832 patients participated in the study, with a mean age of 48.7±16.1 (18-90) years, of whom 244 (29.3%) were elderly and 478 (57.5%) were female.

Regarding the surgical groups of the patients studied, digestive tract surgeries were more prevalent (43.1%), followed by urology (22%) and abdominal wall (11.7%), as shown in Figure 2. There was no significant difference between the two fasting groups studied.

[Fig f1]

**Figure 1 f1:**
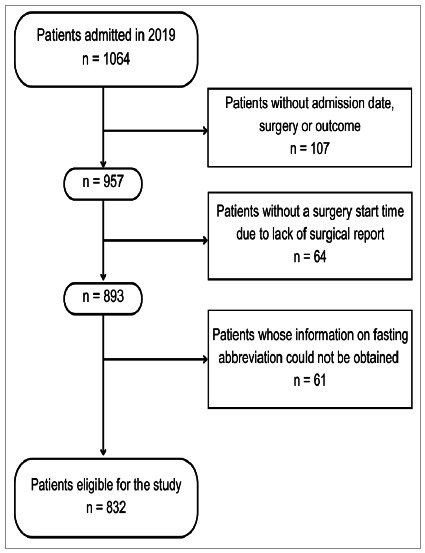
Sample flow diagram, considering inclusion and exclusion criteria.

**Figure 2:**
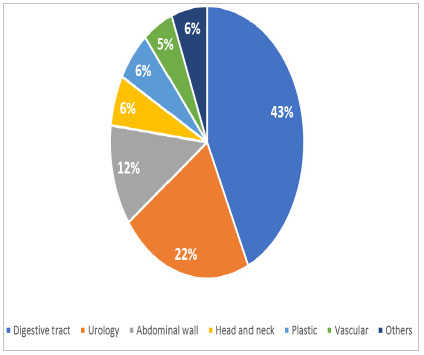
Surgical groups of the patients included in the study.

Of this sample, 678 patients (81.5%) received fasting abbreviation with maltodextrin. The mean length of hospital stay was 3.2 days, with 2.24 in the group with shortened fast and 7.5 days in the group without it (p<0.01). Among the individuals studied, 3.8% had complications, 1% had infection, and mortality was 1.9%. Only 6.5% of the patients received nutritional therapy, either oral, enteral, or parenteral. 

The mean fasting time of the study participants was 357.2 ± 233 (100-1,244) minutes, with 267.92 ± 89.8 (120-605) minutes in the short fasting group and 768.6 ± 247.8 (150-1,244) minutes in the conventional fasting group (p<0.01).

Patients who received a prescription for shortening fasting (intention to treat) had a lower mean cost than those who did not (R$ 3,245.37 ± 4,157.5 vs R$ 10,897.39 ± 16,701.3; p<0.01), as shown in Table 1.

**Table 1:** Hospital costs in relation to fasting abbreviation intention to treat.

Variables	Frequency			
Fasting abbreviation	n (%)	Costs (R$)	IQR 25-75 (R$)	p
Yes	678 (81.5)	3,245.37	1,442.86-2,885.72	(<0.01)
No	154 (18.5)	10,897.39	1,442.86-12,985.74	

Parametric t-tests were unpaired. The statistical significance limit was 5% (p< 0.05).

However, we observed that 393 patients (47.2%) actually fasted for more than five hours, which is the number studied without the intention to treat, as can be seen in Table 2. Likewise, we observed that the costs were significantly higher in the group that effectively fasted for more than five hours.

**Table 2:** Analysis of hospital costs in relation to fasting abbreviation of less than five hours.

Variables	Frequency			
Intention to treat	n (%)	Costs (R$)	SD	p
Fasting less than 5 hours	439 (52.8)	3,284.32	± 4,570.84	<0.001
Fasting longer than 5 hours	393 (47.2)	6,193.83	± 11,414.72	

Parametric t-tests were unpaired. The statistical significance limit was 5% (p< 0.05).

### Univariate analysis

The results of hospital costs and other variables studied can be seen in Table 3. There were significantly more costs associated with males, diabetics, and malnourished individuals.

**Table 3:** Analysis of risk factors associated with preoperative condition and hospital costs of the patients included in the study.

Variables		Frequency n (%)	Costs (R$)	p
Sex	Male	353 (42,4)	5497,58	0,02
	Female	479 (57,6)	4026,73	
Age	Elderly	244 (29,3)	5162,36	0,27
	Non-elderly	588 (70,7)	4439,19	
SAH	Yes	273 (32,8)	5015,66	0,39
	No	559 (67,2)	4473,38	
DM	Yes	81 (9,7)	7837,76	<0.01
	No	751 (90,3)	4307,42	
SGA	A	214 (44,1)	2353,07	<0.01
	B	217 (44,8)	5645,11	
	C	54 (11,1)	20119,88	
Smoking	Yes	89 (10,7)	6241,59	0,06
	No	743 (89,3)	4460,81	
Alcoholism	Yes	72 (8,6)	3727,39	0,34
	No	760 (91,4)	4739,19	

For univariate analysis in continuous data with Gaussian distribution, we used the parametric unpaired t-tests, and for non-homogeneous data, the Mann-Whitney and the Kruskal-Wallis tests, for variables with more than three categories. For qualitative variables, chi-square and Fisher’s exact tests were used. The statistical significance limit was 5% (p< 0.05).

### Multivariate analysis

**Figure 3:**
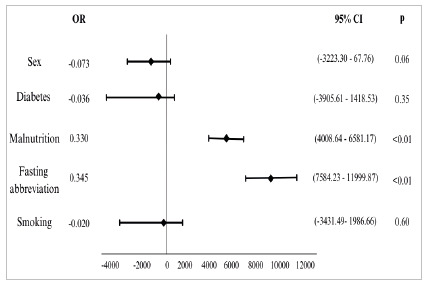
Multivariate analysis of the variables associated with higher costs. Linear regression model including variables that in the univariate analysis presented p<0.20.

As described in the methods section, the following variables were included in the multivariate analysis model: gender, SGA, diabetes mellitus, smoking, and fasting abbreviation. The findings, as shown in Figure 3, were significantly associated with higher costs, malnutrition, and not receiving the preoperative fasting abbreviation protocol (fasting for more than five hours).

## DISCUSSION

The results showed that fasting abbreviation reduced costs in surgical procedures. Several studies that compared costs with or without the ERAS multimodal protocol have shown the same with international currencies such as euros, pounds sterling, or yuan^25^ ^,^ ^26^. 

Although most studies agree that multimodal protocols can reduce costs, the question is about what mechanism might lead to these findings. The hypotheses are of two main factors: reduction in length of hospital stay and reduction of perioperative complications^25^.

Most of the ERAS and ACERTO studies report a reduction in the length of hospital stay^17^ ^,^ ^27^. This can be explained by earlier discharges that reduced the number of resources assigned to patients, thus reducing costs. Some calculations indicate that hospitals save approximately US$ 2,000 per day of reduced length of stay^26^. Another mechanism by which protocols reduce costs is through the reduction of perioperative complications. In this study, this analysis was limited due to the small number of complications, but the effects of multimodal protocols on their reduction have already been documented in the literature^17^ ^,^ ^26^.

Economic analysis is important to ensure the success and durability of multimodal programs, as they require a significant initial investment of resources, whether capital, leadership, or time. Hospital administrators are increasingly experimenting with cost-reducing strategies, and the ACERTO protocol can be implemented safely and effectively.

The data from this study showed that although the preoperative fasting period is generally longer than prescribed, fasting is shorter in patients receiving the abbreviation. The reasons for prolonged preoperative fasting in surgical patients are probably multifactorial, ranging from delays, fear of aspiration on the part of some professionals, changes in procedures times, and an extension of the fasting time prescribed by patients who believe that it could improve their condition^28^.

In addition to the abbreviation for preoperative fasting, another variable that has been shown to be significantly associated with higher cost is malnutrition. According to the ACERTO Guideline for perioperative nutritional interventions^20^, nutritional status interferes with postoperative results, because the more compromised the nutritional status, the higher the risks of morbidity and mortality and, consequently, the higher the hospital costs.

Similar results were found by Mosquera et.al.^29^, in which the length of hospital stay, postoperative complications, and hospital costs were higher in malnourished patients compared with patients with normal nutritional status. 

The impact of hospital malnutrition on health care costs is multifactorial. However, the longer length of stay is the most documented variable in the literature^30^ ^,^ ^31^, as malnourished patients remain hospitalized for longer periods. A longer hospital stay not only has an impact on costs but is also related to the availability of more beds, which are essential in countries with fewer resources, such as Brazil^30^. 

There are inherent limitations to this study, including being from a single institution and cost data not being collected at the individual level. This approach limits the overall applicability but demonstrates the concept that the implementation of the fasting abbreviation in the context of the same surgeons and institutional costs has resulted in a reduction in expenses. 

The greatest limitation of the study, however, is the sample population composed of a heterogeneous variety of surgical procedures, the most prevalent being digestive tract surgeries, followed by urological surgeries. Thus, analyzing such distinct groups together can be a disadvantage. Nonetheless, the surgical groups were analyzed separately (data not shown) and the main results remained unchanged, even if looked at separately.

Thus, the overall results seem to agree that the change from traditional to modern perioperative care, such as reducing preoperative fasting, can reduce hospital costs.

## CONCLUSION

The abbreviation of preoperative fasting reduces hospital costs. Corroborating prolonged fasting, malnutrition also makes hospitalization more expensive. 

This study highlights how adequate preoperative care of hospitalized patients is extremely important in the quality of health care delivery, bringing clinically and economically significant results. In addition, this study highlights that the ACERTO protocol is a valuable intervention for the health system, especially for the Brazilian public health one, as by reducing the length of hospital stay, the available hospital beds are increased, which would benefit more patients who need hospitalization.
